# Relatively effortless listening promotes understanding and recall of medical instructions in older adults

**DOI:** 10.3389/fpsyg.2015.00778

**Published:** 2015-06-09

**Authors:** Roberta M. DiDonato, Aimée M. Surprenant

**Affiliations:** ^1^Cognitive Aging and Memory Lab, Department of Psychology, Memorial University of NewfoundlandSt. John's, NL, Canada; ^2^Speech Language Pathology, Medicine Department, Eastern HealthSt. John's, NL, Canada

**Keywords:** memory, hearing loss, aging, auditory processing, comprehension

## Abstract

Communication success under adverse conditions requires efficient and effective recruitment of both bottom-up (sensori-perceptual) and top-down (cognitive-linguistic) resources to decode the intended auditory-verbal message. Employing these limited capacity resources has been shown to vary across the lifespan, with evidence indicating that younger adults out-perform older adults for both comprehension and memory of the message. This study examined how sources of interference arising from the speaker (message spoken with conversational vs. clear speech technique), the listener (hearing-listening and cognitive-linguistic factors), and the environment (in competing speech babble noise vs. quiet) interact and influence learning and memory performance using more ecologically valid methods than has been done previously. The results suggest that when older adults listened to complex medical prescription instructions with “clear speech,” (presented at audible levels through insertion earphones) their learning efficiency, immediate, and delayed memory performance improved relative to their performance when they listened with a normal conversational speech rate (presented at audible levels in sound field). This better learning and memory performance for clear speech listening was maintained even in the presence of speech babble noise. The finding that there was the largest learning-practice effect on 2nd trial performance in the conversational speech when the clear speech listening condition was first is suggestive of greater experience-dependent perceptual learning or adaptation to the speaker's speech and voice pattern in clear speech. This suggests that experience-dependent perceptual learning plays a role in facilitating the language processing and comprehension of a message and subsequent memory encoding.

## Introduction

Adverse listening conditions that may hinder communication success arise from multiple sources. They may arise from within the speaker (imprecise articulation or accented speech), within the listener (hearing loss or cognitive-linguistic compromise) and/or within the environment (degraded transmission of the communication signal from telecommunication systems) (Mattys et al., 2009, 2012; Mattys and Wiget, 2011). By examining how speaker, listener and environmental sources of interference interact and influence language understanding and communication success, those factors or mechanisms that may also hinder or facilitate learning and memory performance can be identified (McCoy et al., 2005). This could have many practical impacts. First, those components that are most amenable to intervention could be improved in order to affect functional performance of activities of daily living that require communication and memory of important instructions (IADLs). Second, understanding them will advance our knowledge of how age-related changes in sensory-perceptual abilities influence cognitive decline in the older adult and may provide opportunities for prevention.

The primary purpose of this study was to accomplish the following goals: (1) to examine whether a specific type of auditory enhancement, a message spoken with clear speech technique, relative to normal conversational speech results in better learning efficiency, immediate, and delayed memory performance (Bradlow et al., 2003); (2) to investigate whether a distractor (e.g., speech babble noise) decreases learning and memory performance similarly in both the conversational and clear speech listening conditions; and (3) to determine how individual differences in hearing-listening and cognitive-linguistic factors contribute to memory performance. Three sources that contribute to adverse listening conditions were examined: those that arise within the speaker (conversation vs. clear speech), the listener (hearing-listening or cognitive linguistic functioning) and the environment (noise vs. quiet). Further, due to the nature of the design, learning-practice effects were also considered in this study. Specifically it was important to determine if memory performance was influenced as a result of practice with the experimental tasks, specifically for the role of experience-dependent perceptional learning or adaptation to the speaker (Peelle and Wingfield, 2005).

A secondary purpose was to examine this in an ecologically valid manner that captures real-life listening, language comprehension, and memory performance that is pragmatically relevant for many older adults. One motivation to use ecologically valid methods and tasks is to generalize these findings to more typical communication scenarios that require dual-tasking such as learning a task while listening to instructions (Schaefer, 2014). Additionally, as Gilbert et al. (2014) suggested, enhanced speech intelligibility with ecologically valid methods is necessary for examining how speech perception and processing in more naturalistic communicative scenarios influences listening effort and memory in older adults. Another motivation is to address the criticism of cognitive-aging research that uses methods and tasks that are more relevant to university students and less relevant to older adults, particularly when comparing the groups' performance. The criticism is that the older adults' poorer performance could be attributed to reasons unrelated to cognitive-aging decline (older adults view tasks to be patently artificial and therefore are less motivated to perform) (Craik and Bialystok, 2006).

Age-related hearing loss (ARHL) can be defined as a combination of auditory perceptual and auditory processing deficits. These age-related changes in auditory perception and processing have been demonstrated to occur as early as middle age (e.g., 40–57 years old) (Working Group on Speech Understanding and Aging and the Committee on Hearing, Bioacoustics and Biomechanics (CHABA), 1988; Helfer and Vargo, 2009; Wambacq et al., 2009). The etiology of ARHL can be attributed to a combination of the auditory stressors that are acquired throughout the life span (e.g., trauma, noise, and otologic diseases) together with genetically controlled aging processes (CHABA, 1988). Older adults with clinically normal audiograms demonstrate less *dynamic* temporal processing abilities as compared to younger adults with normal hearing (Konkle et al., 1977; Gordon-Salant and Fitzgibbons, 1993). Additionally, a mixed-type hearing loss is also consistent with this definition of ARHL. Therefore, a broader definition of ARHL beyond the audiogram (high frequency sensori-neural hearing loss) was considered for this study, one that incorporates these other aspects of hearing-listening changes that interfere with signal processing for speech understanding (Anderson et al., 2011, 2012; John et al., 2012).

There is evidence that as we age, particularly around the 6th decade of life, our listening abilities are less precise and less efficient compared to younger adults in the 2nd to 3rd decades of life (CHABA, 1988). These age-related hearing-listening changes distort and degrade the stimuli (Rosen, 1992; Gordon-Salant and Fitzgibbons, 1993). These listening difficulties arise from at least three general areas: decreased audibility particularly in the high frequencies disrupting consonant discrimination (Humes, 2008), slowed temporal processing or adaptation (Peelle and Wingfield, 2005) interference with experience-dependent perceptual learning of the speaker's voice and speech pattern, and difficulty segmenting the target from a competing message (e.g., listening in noise). The listening-in-noise difficulty evident in the older adult arises from both domain-specific processes (such as auditory stream segregation) and domain-general cognitive-linguistic processes (such as attention, task switching, inhibition, and monitoring capacity) (Anderson et al., 2012, 2013; Humes et al., 2012; Amichetti et al., 2013).

Furthermore, several studies have shown that even mild hearing loss that has no measurable effect on speech understanding in quiet listening conditions can have substantial effects in noisy or other adverse conditions for both discriminating words (CHABA, 1988), and memory for words recognized (Rabbitt, 1990; Pichora-Fuller et al., 1995; Mattys et al., 2009, 2012; Ng et al., 2013).

The ability to understand spoken language is necessary for functional performance of instructional activities of daily living (IALDs) (e.g., use of medical instructions for medical adherence). Fundamental to comprehension and learning of an auditory-verbal message are sufficiently intact auditory perceptual-processing abilities and cognitive-linguistic functioning. These bottom-up (auditory perceptual-processing) and top-down (cognitive-linguistic) processes need to be efficiently recruited to effectively decode the message for communication success. Both implicit and explicit recruitment of these limited-capacity resources (Kahneman, 1973), perhaps as compensation (Bäckman and Dixon, 1992; Rönnberg et al., 2010; Wild et al., 2012) have been demonstrated to promote ease of language understanding in sub-optimal or adverse communication scenarios.

Rönnberg et al. (2008) used a working memory model for Ease of Language Understanding (ELU) to explain how perceptual processes interact with cognitive processes for understanding. They proposed that it is the relative fidelity of the speech message that allows for the ease or automaticity of the match between the upstream sub-lexical features (phonology) and the target in the lexicon. Thus, when the fidelity is optimal, the match with the target occurs, at the exclusion of other competing targets in the lexicon, more rapidly and automatically due to implicit processes. When the fidelity of the message is low or suboptimal, the automatic matching processes of the sub-lexical features to the target in the lexicon is unsuccessful, resulting in a mismatch. The ELU model suggests that controlled processes are then required such that the sub-lexical, lexical, and semantic and conceptual representations from long-term memory are needed to further decode the speech signal. The match then occurs by way of explicit processes (Rönnberg et al., 2008, 2013). Thus, the re-allocation of explicit cognitive-linguistic resources for decoding of the speech signal results in fewer resources available for the learning and recall of the materials heard. Under optimal listening conditions fewer explicit resources are needed for comprehension, presumably because the perceptual features more closely match the listener's sub-lexical and lexical features in long-term memory. Optimizing the fidelity of the spoken message allows for more rapid and automatic-implicit perceptual learning of the speaker (Rudner et al., 2009) and more cognitive-linguistic resources will be available for comprehension, learning, and recall of the message (Wingfield et al., 1985, 1999, 2006; Wingfield and Ducharme, 1999).

One method to optimize the listening situation is to increase the fidelity of the speech message by using a style of speaking that increases the speech intelligibility. The “clear speech technique” is one in which the talker is instructed to produce the speech as if speaking to someone who is either hearing impaired or to one who is not a native speaker of the language (Ferguson and Kewley-Port, 2007). These were the instructions provided to the male speaker who produced the stimuli for our study. This “clear speech” technique resulted in an average speaking rate of 145 syllables per minute (spm). Relative to the original-conversational rate of the vignettes (192.5 spm), the clear speech rate was on the slower end of the normal speech rate (Goldman-Eisler, 1968); consistent with other studies that use this technique (Ferguson, 2012).

In addition to a slower rate of speech, other acoustic dimensions change by using the “clear speech” technique. The acoustic characteristics that give clear speech its intelligibility benefit are increased duration of vowels, longer and more frequent pauses, a larger consonant-vowel ratio, increased size of vowel space, decreased alveolar flapping, increased stop-plosive release, more variable voice fundamental frequency (F0), and greater variability in vocal intensity (Bradlow et al., 2003; Ferguson and Kewley-Port, 2007).

Although the use of clear speech has been demonstrated to enhance intelligibility of word and sentence discrimination in younger and older adults with and without hearing loss (Picheny et al., 1985; Ferguson, 2012) less is understood regarding its role for facilitating memory encoding. Gilbert et al. (2014) investigated intelligibility and recognition memory in noise for conversational and clear speech recorded in quiet and in response to the environmental noise (noise adapted speech-NAS) in young normal hearing adults. Results demonstrated that improved intelligibility for clear relative to conversational speech in noise improved recognition memory and that the NAS speech further enhanced intelligibility and recognition memory. Gilbert et al. (2014) concluded that naturalistic methods that simulate real-world communicative conditions for enhancing speech intelligibility have a role in improving speech recognition, comprehension, and memory performance in younger adults and may improve memory abilities for older adults.

Both sensory deficits (such as hearing loss) and cognitive impairments (such as memory difficulties) increase as a function of age and are highly correlated (Baltes and Lindenberger, 1997). In a comprehensive review of the literature, Schneider and Pichora-Fuller (2000) discussed a number of ways in which these sensory and cognitive declines could be related. They suggested that poor memory performance could be partially attributed to unclear and/or distorted perceptual information delivered to the cognitive/memory processes; the so-called “information-degradation hypothesis” (Schneider and Pichora-Fuller, 2000). In addition, several researchers (Rabbitt, 1968, 1990; Surprenant, 1999, 2007; Wingfield et al., 2005, 2006; Stewart and Wingfield, 2009; Tun et al., 2009; Baldwin and Ash, 2011) have argued that perceptual effort has an effect on cognitive resources with concomitant influences on memory performance. This is often referred to as the “effortfulness hypothesis.”

According to the effortfulness hypothesis, if listening effort for decoding the verbal message comes at the cost of cognitive resources that would otherwise be shared with the secondary task of encoding information into memory, then decreasing listening effort should result in improved learning and memory performance. Further, those individuals with greater capacity in hearing-listening and cognitive–linguistic abilities would theoretically have more resources (Kahneman, 1973) to share between the two tasks (Rabbitt, 1968, 1990). Therefore, in order to determine how these bottom up and top down resources contributed to memory performance it was first necessary to examine the participant's unique abilities in hearing-listening and cognitive-linguistic functioning. Then, how these individual variables (hearing and cognition abilities) contribute to the memory performance by listening condition (conversational and clear) and by group (Quiet and Noise) can be examined.

In this study, we recruited older adults with a range from normal-to-moderately impaired hearing-listening abilities. They listened to medical instructions either in quiet or in the presence of background babble. Half of the sentences were presented in conversational speech and half in clear speech. The listeners were asked to repeat the stimuli as precisely as they could after each trial of listening. After a filled delay they were asked to recall all the information that they heard. We examined learning efficiency defined as the averaged amount of the stimuli repeated over the four trials to learn; immediate memory as the total of items repeated immediately; and the delayed memory as the total of items recalled after a delay period. We compared learning and memory performance within subjects for the two listening conditions (clear and conversational) and between subjects for the competition (quiet and noise). In addition, we measured the individual's hearing-listening and cognitive-linguistic abilities to determine how these unique characteristics may have influenced the delayed memory performance in the two listening conditions for the two groups.

For theoretical and practical reasons, we examined how quickly the participant was able to learn the passages, how much they discriminated for immediate repetition and how much of the message they encoded for later free-recall. Theoretically, the question is whether these learning and memory processes in older adults are differentially affected by the change in listening condition. The intention is to identify the dissociable memory processing components that potentially contribute to a decline in memory for older adults (Salthouse, 2010).

Zacks et al. (2000) summarized the theoretical orientations in memory and aging and described three areas that differentiate the younger from the older adult; limited resources, processing speed, and inhibitory control.

Older adults are more limited in essential resources or self-initiated processing both at encoding and retrieval (Hasher and Zacks, 1979; Light, 1991; Craik et al., 1995). Relative to younger adults, older adults are more negatively affected by free-recall tasks, which require a higher degree of self-initiated processes. For the present study, the type of memory task chosen was free-recall. If the experimental manipulation to enhance the auditory stimuli improves the older adult's free-recall performance relative to conversational speech it will suggest that the age differences in free-recall, consistently reported by other authors (Salthouse, 2010), may be partially attributed to the effort in listening which consumes those same resources.

Older adults process information slower than younger adults (Park et al., 1996; Salthouse, 1996; Verhaeghen and Salthouse, 1997). According to Salthouse (1996) in situations in which time is restricted, the time required for the memory processes to rehearse or elaborately encode may be compromised by earlier processes, consuming the total time available to perform the task.

In relation to the present study, auditory enhancement (clear speech), which facilitates more timely and automatic processes for auditory perception and processing of the message, should free up time for those memory processes. In this way the auditory enhancements may facilitate faster perceptual learning or adaptation to the speaker's pattern. A larger learning effect (better learning or memory performance on 2nd trial of a task) indicates that the more automatic and timely auditory processing of the message for comprehension has allowed for more time available to rehearse or elaborately encode information for later recall. If learning effects differ by listening condition for the older adults, this finding suggests that some of the age-related slowing may be attributed to differences in perceptual learning of the speaker's pattern.

Older adults have less inhibitory control particularly for attention to the relevant contents of working memory. The increased mental clutter due to poorer inhibitory control increases the likelihood for sources of interference, both at encoding and retrieval (Hasher and Zacks, 1988; Zacks and Hasher, 1994, 1997; Hasher et al., 1999). In relation to the present study, the older adult with ARHL may experience an increase in mental clutter from the perceptual and lexical processing loads (Mattys and Scharenborg, 2014). Inhibiting this “noise” and maintaining attention to the task for both comprehension of the message and encoding into memory requires greater inhibitory control (or executive function) and working memory capacity for successful performance. In this way, the individual's executive control, working memory, and short-term memory is taxed more in adverse listening conditions relative to easier listening. Relevant to this study, those individuals with strengths in inhibitory control and working memory capacity should demonstrate better learning and memory performance, particularly for adverse listening conditions in which these resources are strained.

Both the ELU and the effortfulness hypotheses were considered for this study. According to the effortfulness hypothesis first described by Rabbitt (1968) and subsequently others (Tun et al., 2002, 2009; McCoy et al., 2005), while listening to typically spoken messages in degraded conditions, cognitive-linguistic resources are re-allocated for deciphering the message. This re-allocation of resources comes at the cost of those same resources for learning and memory encoding (Kahneman, 1973). The stimuli here were constructed in such a way as to optimize the auditory processing of the verbal message. The expectation is that the enhanced stimuli “clear speech technique” will mitigate those aspects of age-related hearing that interfere with communication success by reducing the perceptual, lexical, and cognitive loads (Mattys et al., 2012). In so doing, enhanced listening will free up those resources that are required for elaborate encoding for learning and remembering the passages.

Similarly, according to the ELU (Rönnberg et al., 2008), if the match between the stimuli and the long-term representation of the target in memory is automatic, then fewer explicit resources will be required for understanding the message. If we can enhance the clarity of the speech by using a style of speaking that promotes an intelligibility benefit, these same explicit cognitive-linguistic resources should become available for perceptual learning, comprehension, and elaborate encoding for later recall. Both of these hypotheses suggest that easier auditory processing of the message results in easier learning and recall. Also the suggestion is that resources for listening, learning, and remembering processes are limited and must be shared or re-allocated as needed (Gilbert et al., 2014).

If the hypotheses are confirmed, there should be a main effect of listening condition: Relative to conversational speech, enhanced listening will result in more efficient learning and better immediate and delayed memory performance. If the irrelevant speech-babble noise further interferes with processing of the targeted message then there will be a main effect of speech babble noise and an interaction of listening condition and group (Quiet vs. Noise). If found, the difference in memory performance between the two groups could be attributed to either energetic masking (Heinrich et al., 2008) of the stimuli, the noise covers up part of the sub-lexical acoustic information of the target; and/or a distractor effect, the noise distracts the listener's attention from the target (Lavie and DeFockert, 2003; Lavie, 2005; Mattys et al., 2009). In both scenarios, re-allocation of explicit cognitive-linguistic resources are required to “fill in” for what was missed to understand the message, while inhibiting the to-be-ignored background and maintaining focus for processing of the ongoing message.

## Materials and methods

### Participants

Ethics clearance was obtained from Memorial University's Interdisciplinary Committee on Ethics in Human Research (ICEHR) in accordance with the Tri-Council Policy Statement on Ethical Conduct involving Humans. Inclusion criteria: community dwelling-healthy older adults 55+ years old. Exclusion criteria: known medical events that may affect cognition (e.g., cardiovascular event, neurological event, or disease), failed cognitive screening, insufficient corrected vision for performing the experiment, and hearing loss that exceeded the capacity of the speakers (90 dBA). To determine the sample size required to detect a small effect size we used G^*^Power 3.1 (Faul et al., 2007) (Input: Effect size f = 0.26 α error probability = 0.05, Power (1-β error probability) = 0.95, Number of groups = 2, Number of measurements = 3, Correlations among repeated measures (learning efficiency, immediate, and delayed memory) = 0.5, Non-sphericity correction ε = 1. Output: Non-centrality parameter λ = 16.22, Critical F = 3.17, Numerator df = 2.0, Denominator df = 76.0). This suggested a total sample size of 40 participants. We over-recruited by 20% (e.g., 48 participants recruited) to account for attrition.

**Figure 1:**
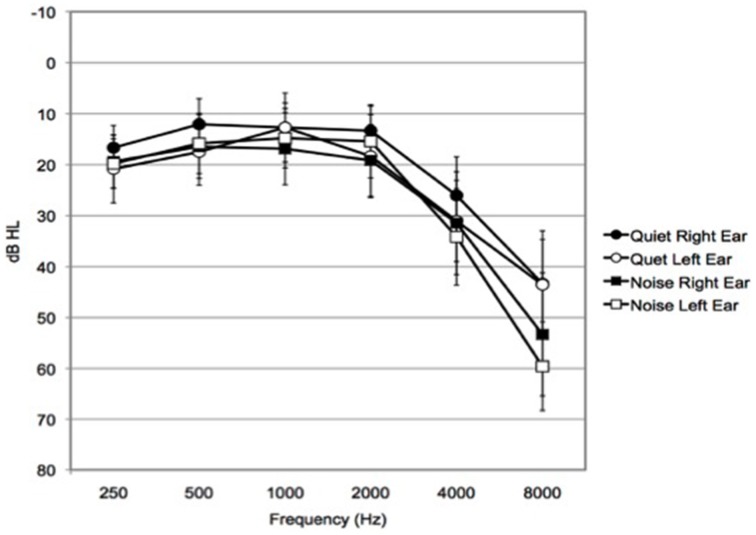
**Mean audiogram profile**. Hearing thresholds of all participants in this study. Mean audiogram profile of Quiet group right ear and Quiet group left ear (*n* = 24), Noise group right ear and Noise group left ear (*n* = 24). Bars represent 95% confidence intervals.

Forty-eight older adults were recruited to participate and were randomly assigned to either the Quiet (*n* = 24, 14 females) or Noise (*n* = 24, 12 females) group. This was accomplished by first generating a counterbalanced and randomized list for the two groups and the eight different orders for completing the experiment, then the participant was allocated to the pre-randomized group/order condition sequentially. Three participants wore hearing aids, two in the Quiet, and one in the Noise group. (See Table 1 for demographic, hearing and cognitive characteristics means and standard deviations; see Figure 1 for audiogram data.) Participants received $10 an hour for their participation.

**Table 1:** **Demographics, Hearing, and Cognitive Characteristics**.

**Characteristics**	**Quiet**		**Noise**		**Range**
	***M***	***(SD)***	***M***	***(SD)***	***Min/Max***
**DEMOGRAPHIC VARIABLES**					
Age (years)	65.29	(6.16)	64.79	(6.94)	55/81
Education^a^	3.71	(1.04)	3.92	(1.06)	2/5
Health^b^	3.88	(0.74)	4.00	(0.83)	3/5
**HEARING CHARACTERISTICS**					
QuickSIN^c^	1.33	(1.39)	2.38	(1.64)	(−)1/(+)7^*^
HHIA Survey^d^	8.92	(12.62)	6.92	(10.27)	0/52
RPTA4 (dBHL)	16.04	(11.25)	20.99	(16.54)	(−)2.50/(+) 57.5
LPTA4 (dBHL)	19.90	(14.60)	20.05	(14.25)	(+)3.75/(+)65
Musicianship^e^	2.21	(2.67)	1.46	(2.13)	0/8
**COGNITIVE CHARACTERISTICS**					
FAS (words)^f^	43.04	(12.17)	42.63	(13.87)	17/73
BNT (words)^g^	56.79	(3.74)	55.00	(8.02)	23/60
Digits Back^h^	5.00	(0.93)	4.16	(1.30)	2/7^*^
L-Span (letters)^i^	18.13	(10.07)	17.04	(9.06)	0/42

*Means and Standard Deviations*.

a *Education: self-reported category: 1, some High school; 2, High School; 3, some University/College; 4, University/college degree; 5, Graduate/professional degree*.

b *Health: self-reported category: 1, very poor; 2, poor; 3, good; 4, very good; 5, excellent*.

c *QuickSIN, Quick Speech-in-Noise measurement that provides a signal-to-noise ratio expressed as dB SNR loss, higher numbers indicate poorer abilities. Normal value, < +3 dB SNR loss (Killion, 2002)*.

d *HHIA-Hearing Handicap Inventory for Adults: self-assessment; higher scores indicate greater perception of hearing handicap*.

e *Musicianship: interval scale 0–10 points (higher number reflects greater musicianship experience: 0, no music; 3, some previous music experience in past; 5, some past and current music; 10, full musician)*.

f *FAS- verbal fluency-executive function task, higher number of words generated is better performance*.

g *BNT-Boston Naming Test, higher number of pictures correctly named is better performance*.

h *BackDigit Span-backwards digit span, mean number of digits reported for final 10 trials, higher number is better performance*.

i *L-Span-Listening span, the sum total of letters recalled for each list length recalled with 100%. Larger number is better performance*.

* *p < 0.05*.

### Preliminary measures

The purpose of these measures was to determine if an individual should be excluded from the study. No participant was excluded from the experiment based on the measures of vision, hearing, or the cognitive screening (e.g., passing score is >23) (Crum et al., 1993) the scores ranged from 27 to 30 on the Mini-Mental Status Examination (MMSE) (Folstein et al., 1975).

The following hearing-listening and cognitive-linguistic measures were obtained for all participants, the rationale for these measures and the standardized methods used are described in greater detail elsewhere (DiDonato, 2014).

#### Hearing-listening measures

Audiometric tests were conducted in a single-walled sound attenuated chamber using a *Grason Stadler Instruments* Audiometer (GSI-61), Telephonics TDH50P headphones, E.a.r.Tone™ 3A insert earphones and free-field speakers calibrated to specification (American National Standards Institute ANSI, 2004). Standardized procedures with the TDH50P headphones were used to obtain pure-tone hearing thresholds for right (R) and left (L) ear. Pure tone average (PTA4) is the average of 0.5, 1, 2, and 4 kHz in dB HL (Katz, 1978). PTA4 was the metric used to indicate degree of auditory acuity deficit consistent with the WHO definition (PTA4 greater than 25 dB HL) (World Health Organization Prevention of Blindness and Deafness (PBD) Program, 2014). Speech Reception Threshold (SRT) is the threshold in dB at which one can repeat a closed set of words with 50% consistency (Newby, 1979). The Phonetically balanced (PB) max-most comfortable loudness level (PB max-MCL) is the intensity level measured in decibels in Hearing Level (dB HL), for which the participants achieved the highest accuracy for repeating phonetically-balanced (PB) word lists (Newby, 1979). The SRT and PB max-MCL were used to calculate the sensation level in which participants experienced the stimuli.

The Quick Speech-In-Noise test (QuickSIN): Etymotic Research, Elk Grove, IL; (Killion et al., 2004) is a standardized assessment of the ability to repeat/recall sentences from a target speaker (a female voice) in the presence of multi-talker babble at various levels of speech-in-noise ratios (SNRs). The target sentences were routed through the GSI-61 audiometer's external channel at 70 dB HL via the free-field speaker (Killion, 2002). The score is the signal-to-noise ratio (SNR), in decibels (dB), in which the listener recognizes the speech target correctly with 50% accuracy. A score of +7 dB SNR loss on the QuickSIN indicates that the individual needs the signal to be 7 dB louder than the competing speech noise in order to identify the sentences with 50% accuracy. Higher values reflect poorer listening-in-noise ability. The Hearing Handicap Inventory for Adults HHIA (Newman et al., 1991) is a standardized and normed self-assessment used clinically to determine the individual's self-perception of the degree to which they experience a handicap due to hearing loss (adapted from Hearing Handicap Inventory for the Elderly, HHIE (Ventry and Weinstein, 1982). The questions reflect both the social/situational and emotional consequences of hearing loss. The individual's response is yes (4 points), sometimes (2 points), or no (0 points). The score is the sum total of all the responses. A higher value reflects a greater perception of hearing handicap.

A musicianship score was calculated based on the responses to the demographic questionnaire regarding musical experience. The demographic questionnaire also included questions regarding age, education, occupation, health, medication use, and language(s) spoken (see Appendix A in supplementary Material). The musicianship classification score created for this study was an interval scale in which a higher value reflected more experience with music. Participants answered questions regarding exposure to music, age of onset of formal training, duration in years of musical performance, and the extent to which they were engaged in musical practice (e.g., hours/days per week). These questions were consistent with other studies that examine musical training and its relationship with auditory perceptual and processing abilities in behavioral and electrophysiological studies (Kraus and Chandrasekaran, 2010; Zendel and Alain, 2012, 2013). A composite score was calculated so that participants had a musicianship score from 0 to 10. A minimum score of 0 reflected no early music education, no formal lessons, and no instrumental or vocal performance presently or in the past. Maximum score of 10 reflected those who identify themselves as a musician (not necessarily professionally), started music education by 10 years of age or younger, had been musically active throughout their lifetime, had performed 12 years or greater, and those who currently perform on average at a minimum of 6 h weekly.

#### Cognitive-linguistic measures

Listening span (L-span) is a working memory (WM) task that is similar to the reading span measure (Daneman and Carpenter, 1980). The rationale for using a WM span task in this study was that this type of span task is highly predictive for complex cognitive behaviors across domains such as understanding spoken language and reading comprehension (Just and Carpenter, 1992; St Clair-Thompson and Sykes, 2010). Participants heard a sentence and had to indicate whether the last word in the sentence was predictable or not predictable (mouse-click on the respective boxes on the computer screen). At the same time that they heard the sentence, they saw a letter on the computer screen. They were instructed to attend to the letters presented and after a series of sentences and letters, were cued to recreate the letter sequence in order. The sum total of all the list lengths, which were correctly recalled, is the score. Higher scores reflect better working memory. Backward digit span (Wechsler, 1981) is a task that correlates with other measures of cognitive function such as working memory capacity, but not so strongly that it measures the same construct (Conway et al., 2005; St Clair-Thompson, 2010). Participants heard lists of digits and recreated them in reverse order. The score reflects the mean number of digits recreated in reverse order for the final 10 trials. Boston Naming Test (BNT) is a subtest of the Boston Diagnostic Aphasia Examination (Kaplan et al., 2001). The BNT is a standardized and normed confrontation picture-naming task. Participants name 60 line drawings, 1 point for each correctly named item. The BNT has been found to have good internal consistency and high reliability (Goodglass et al., 2001). Verbal fluency measure (FAS) correlates with other metrics that measure executive function. Scores reflect the individual's cognitive flexibility, inhibition and response generation (Mueller and Dollaghan, 2013). Participants generate as many words as possible beginning with the letter “F,” “A,” and “S,” given 1 min for each letter. The score is the total number of words generated.

### Comparing groups on demographic, hearing, and cognitive measures

There were no differences on demographic, hearing, and cognitive measures between the competition groups (Quiet/Noise) by ANOVA or Mann-Whitney *U*-tests (where appropriate) (smallest *p* > 0.23) except on the QuickSIN, *F*_(1, 47)_ = 5.65, *p* = 0.02, and Backward digit span, *F*_(1, 38)_ = 5.36, *p* = 0.03. The Quiet group demonstrated better listening-in-noise abilities, *M*_Quiet_ = 1.33 dB, *SD* = 1.39 dB, compared to the Noise group *M*_Noise_ = 2.38 dB, *SD* = 1.64 dB. The Quiet group demonstrated longer backward digit span values (*M*_Quiet_ = 5.00, *SD* = 0.93), compared to the Noise group (*M*_Noise_ = 4.16, *SD* = 1.30). Due to an error in the program there were nine backward digits scores that had been incorrectly calculated (5 Quiet, 4 Noise); these values were not entered in the analysis for this measure. (Table 1).

There were unexpected *a priori* differences between the groups. If differences exist between the two competition groups for the learning and memory performance in the two listening conditions, these variables must be considered and understood in terms of their impact. The Quiet group's better listening-in-noise and short-term memory abilities could result in better learning and memory performance for the two listening conditions independent of the lack of noise (i.e., erroneously concluding that the noise interfered with performance). However, no main effect of group or interaction would suggest that these differences did not influence the result.

### The auditory-verbal stimuli

Fictionalized medical prescription vignettes were created. The vignettes were thematic in nature and described the multiple steps needed to use specific medical prescriptions (see Appendix B in Supplementary Material for the two vignettes: medipatch and puffer-inhaler and training item). These vignettes were matched on many linguistic and non-linguistic aspects of speech to equate them as much as possible on the complexity of the stimuli, while at the same time maintaining their ecological validity (see Table 2). Both sets of prescription instructions comprised 10 sentences, with 37 critical units (CU) to report. The 37 CU were the content words within each phrase that carried the most important salient meaning for the practical purpose of using these fictional medications. Critical units may be a single word, compound word, or multiple words (e.g., breathe out, out of reach). The distribution of the CU throughout the vignette was arranged so that each third of the vignettes had similar numbers and distribution of items to recall. The two vignettes were spoken at their original-conversational rate, 192.5 (spm) and then these same vignettes were spoken using a slower hyper-articulated “clear speech” technique, (145 spm) (Baker and Bradlow, 2009).

**Table 2:** **Linguistic aspects of the vignettes**.

	**Medipatch**	**Puffer**
**LINGUISTIC FEATURES**		
Total words + (carrier)	100 (15)	89 (24)
Function words	25	26
Content words	75	63
#Syllables CU	73	73
Max #syllables in sentence	21	21
Min #syllables in sentence	3	4
Imperative phrases	11	12
Total #sentences (units)	10 (37)	10 (37)

**Figure 2:**
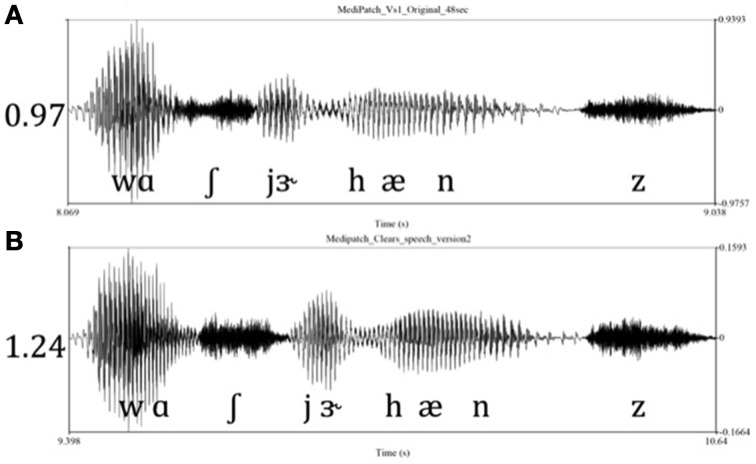
**The Praat waveforms: Two listening conditions**. The waveforms depict the phrase “wash your hands” from the medipatch vignette. The two listening conditions: **(A)** 0.97 s, original format, conversational speech technique (196 spm); **(B)** 1.24 s, spoken with clear speech technique (152 spm). Note in clear speech, the temporal-spectral enhancement can be appreciated by the increased durations of the vowels and increased amplitudes of the waveform.

The *clear speech* and the *conversational speech* vignettes in this experiment were subjected to acoustic analysis using Praat version 5.3.63 (Boersma and Weenink, 2014). Similar to Bradlow et al. (2003), total sentence duration, total number of pauses, average pause duration, F0 mean (Hz), F0 range (Hz), and the average vowel space range in F1 (mels) and F2 (mels) were examined. To calculate the vowel space in mels, the frequency (Hz) was converted to the perceptually motivated mel scale according to the equation by Fant (1973). Similar to Bradlow et al. (2003), when the speaker used a “clear speech” technique there was an increase in the overall duration, the number of pauses, a change in F0 mean and range, and increase in vowel space relative to when the conversational style speech technique was used. Thus, the clear speech vignettes reflect a temporal-spectral enhancement relative to the conversational speech vignettes (see Table 3 for the characteristics of each vignette; Figure 2 for Praat waveform). Avid Pro-tools 8.0.5 was used to manipulate the original sound files to ensure that the recordings were equated for loudness [root mean squared (RMS) amplitude] throughout the passages.

**Table 3:** **Acoustic Characteristics of Conversational (Conv.) and Clear speech**.

**Acoustic Measurement**	**Medipatch**			**Puffer**		
	**Conv**.	**Clear**	**Difference**	**Conv**.	**Clear**	**Difference**
Avg. passage duration (s)	48.30	62.20	13.90	47.00	64.00	17.00
Total # of pauses	12.00	18.00	6.00	10.00	25.00	15.00
Avg. pause duration(ms)	7.60	7.50	0.10	7.10	9.50	2.40
F0 mean (Hz)	113.36	126.02	12.66	114.05	121.84	7.79
Vowel space F1 (mels)	748.02	775.53	27.51	655.32	690.49	35.17
F0 range (Hz)	233.25	308.00	74.74	317.22	356.39	39.17
Vowel space F2 (mels)	1368.41	1426.52	58.11	1442.81	1517.00	74.19

### Research design

There was one between-subjects variable, competition (Quiet vs. Noise) and two within-subjects variables, listening condition (conversational vs. clear speech), and time of memory recall (immediate vs. delayed). This study used a modification of the learn-relearn paradigm (Keisler and Willingham, 2007). Participants listened to, immediately repeated what they had heard (immediate memory), and learned the vignettes as precisely as they could over a series of trials (learning efficiency). They then recalled the vignettes after the completion of 20 min of interference/filler tasks (delayed memory). The participants completed the study in two sessions on two separate days. In the first session they completed the vision screening, audiometric tests and the listening span (L-span). In the second session they completed the experiment as well as the other measures of hearing-listening and cognitive-linguistic abilities (included in the interference/filler task sets A and B).

**Figure 3:**
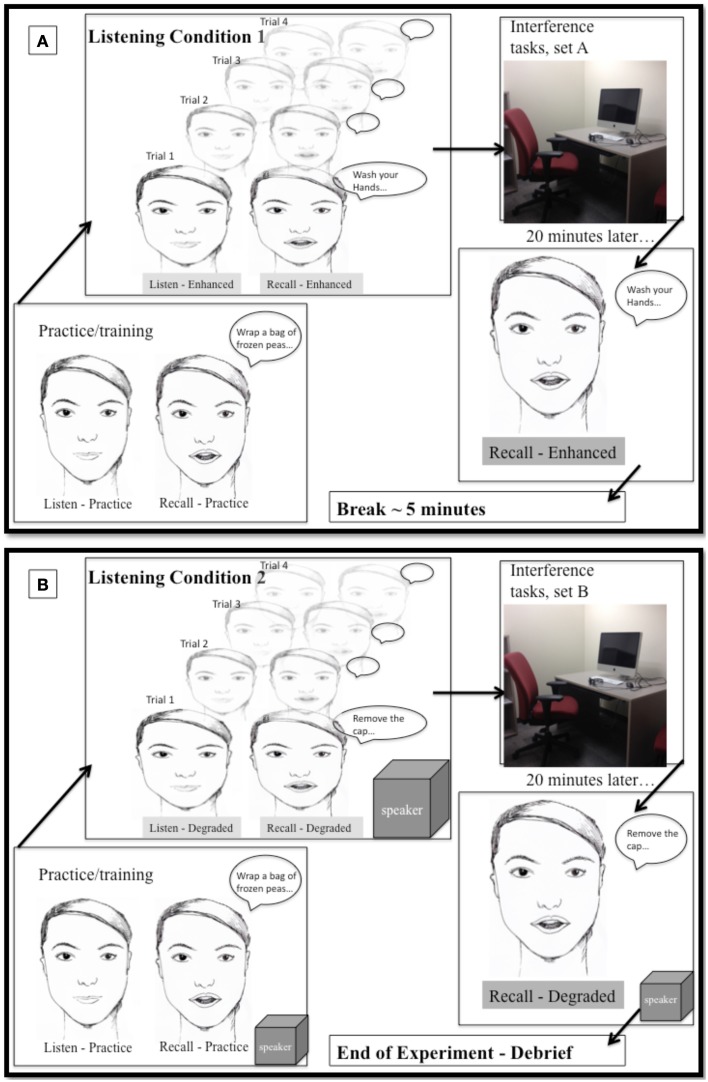
**Illustration of procedures for experiment (second session). (A)** Top panel: Listening condition 1, enhanced (clear speech), via insertion earphones. Participant instructed and practice session. Trials of listening and recall × 4. Move to experiment room for 20 min of interference/filler tasks (set A). Move back to sound booth for delayed recall. Five min break. **(B)** Lower panel: Listening condition 2, degraded (conversational speech) via speaker. Participant re-instructed and practice session repeated. Trials of listening and recall × 4. Move to experiment room for 20 min of interference/filler tasks (set B). Move back to sound booth for delayed recall, end of experiment, debriefing.

Each participant listened to two passages (medipatch and puffer), one spoken with conversational and one in clear speech listening conditions, and all preliminary measures and filler/interference tasks (set A and set B). This resulted in eight different combinations of order conditions. The order in which participants performed the listening conditions, passages, or tasks (set A and B) was counterbalanced and participants were randomly assigned to one of the order conditions. An example of one of the orders is *EmA/DpB*. Figure 3 illustrates the procedures for the second session, when the participant performed the experiment in two listening conditions. In this example, the participant experienced the relatively *Enhanced* listening condition first (clear speech through insertion ear phones) with the *medipatch* passage, completed the interference/filler *tasks set A*. At completion of the timer the participant then returned to the sound booth to recall the medipatch passage. There was a 5-min break (/) between the first and second listening condition. Then the participant experienced the second listening condition, the relatively *Degraded* listening condition (conversational speech through the speaker in sound field) with the *puffer-inhaler* passage, completed the interference/filler *task set B*. Again at completion of the timer the participant returned to the sound booth to recall the puffer-inhaler passage.

Filler/interference tasks. The tasks had two purposes: (1) to provide a delay between listening and delayed recall and a filler activity; and (2) to assess participants on various cognitive and linguistic measures that were later used in the correlation analyses to examine the individual differences in relationship to memory performance. The tasks within each set were administered in the same order. Set A included the (FAS), the backward digit span task, the Philadelphia naming test items 1–87 (Roach et al., 1996), and a demographic questionnaire. Set B included the Philadelphia naming test items 88–175, the BNT, the MMSE, and the HHIA.

There were three dependent measures that were obtained for the two listening conditions as follows: *Learning efficiency* was operationally defined as the mean number of CU learned per trial, calculated using the total sum of the number of CU reported at each of the four trials of learning divided by the number of trials (4). In this way there was a single value for the learning efficiency during the conversational listening, and a single value for the learning efficiency during the clear condition. *Immediate memory* was operationally defined as the sum total of the CU that had been reported during any of the learning trials for that listening condition, to the maximum of a possible total of 37 units (e.g., 1st trial (15) reported CU, plus 2nd trial (5) new CU, plus 3rd trial (3) new CU, plus 4th trial (1) new additional units = 24 CU recalled immediately for that listening condition). *Delayed memory* was operationally defined as the total number of reported CU after the filler tasks for that listening condition, to the maximum of 37 CU.

### Instructions

Participants were informed of the experimental tasks with a written script (see Appendix C in Supplementary Material) that was read aloud to them, while they read along. Answers to questions and redirections to the written instructions were provided prior to and during the training/practice item. They were instructed that they would have multiple trials (4) to learn each vignette and to repeat all that they had heard and remembered after each trial of listening. Participants were instructed that gist reporting was acceptable but were encouraged to use as close to verbatim as possible. The participants were not under any time constraint. Responses were spoken aloud and the responses were audio-recorded. Each trial of listening and then recall of the vignette was recorded into GarageBand '11 on a Macintosh computer for later transcription and off-line scoring. A single research assistant blinded to the listening condition/competition group coded the data.

A training item was created so that participants could understand the nature of the task with feedback provided during the training task, and to confirm that the intensity level determined during the audiometric testing as PB max-MCL was comfortably loud but not too loud. After completion of the training/practice the participant was reminded to perform the experiment as they had just done during the training.

### Presentation of the auditory condition

The stimuli were routed from a MacBook Pro computer via Apogee One, a studio quality USB music interface, to the auxiliary channels of the GSI-61 to the transducers (insert earphones or free-field speaker). The intensity level was set at each individual participant's PB max-MCL obtained during the audiometric testing. This individualized audibility level is consistent with an intensity level that reflects their best performance for discriminating and repeating a list of open-set words in quiet in a sound attenuated chamber.

Despite the advantage of using MCL in dB HL (see DiDonato, 2014), the actual sensation levels or hearing levels for the presentation of the stimuli may have varied by group. Therefore, the sensation level that the participants experienced was calculated for all participants in each group by subtracting the Speech Reception Threshold in dB from the MCL in dB HL, which indicates the sensation level in dB SL. There were no differences between the competition groups (Quiet/Noise) by ANOVA for the sensation level presentation, *F*_(1, 47)_ = 2.98, *p* = 0.09 or for the MCL in dB HL, *F*_(1, 47)_ = 0.96, *p* = 0.33 (see Table 4).

**Table 4:** **Intensity level of stimuli presentation**.

	**Quiet**		**Noise**	
	***M***	***(SD)***	***M***	***(SD)***
Sensation Level dB SL	45.63	(6.81)	41.88	(8.18)
MCL in dB HL	58.96	(6.08)	61.04	(8.47)

#### Conversational speech listening condition

The conversational speech was presented binaurally via a free-field speaker calibrated to a 1 kHz tone. Participants who wore hearing aids did so for this listening condition only. The free-field presentation was used for this listening condition to mimic listening in natural listening environments. All participants were seated and positioned 1 meter distance and 0 degree azimuth to the speaker. *The Noise group*. The conversational speech vignette and competing speech babble noise at +5 dB SNR were routed to the speaker. *The Quiet group*. The conversational speech vignette was routed to the speaker in quiet.

#### Clear speech listening condition

The clear speech stimuli were presented binaurally via disposable 3A E.A.R.tone™ insert earphones. This was intended to further enhance listening by providing optimized signal-to-noise (SNR) benefit. This was done to simulate enhancements for listening by optimizing SNR benefit easily captured in the natural environment (i.e., heard with either a personal FM system, head phones, or through a looped hearing aid). The reality of an SNR benefit of the stimuli in Quiet with the insert earphones in an anechoic sound-attenuated chamber would be much less but perhaps not zero. Additionally, since the clear speech signal and the noise were transduced via the insert earphones simultaneously the SNR benefit would have been nullified for the Noise group. *The Noise group*. The clear speech vignette and competing speech babble noise at +5 dB SNR were presented simultaneously to the insert earphones binaurally. *The Quiet group*. The clear speech vignette was presented without speech babble noise to the insert earphones binaurally.

## Results

To determine the consistency and accuracy of the coding of the participant sound files, one research assistant, blinded to the listening condition, coded all the participant files and then re-coded 21% of the total of the files randomly selected from the experiment. Intra-rater reliabilities for coding of blinded scoring were assessed using intra-class correlation coefficient (ICC) with a two-way mixed effects model and absolute agreement type (Shrout and Fleiss, 1979). The ICC for single measures for the reported-recalled CU for each trial was 0.98. An ICC value between 0.75 and 1.00 is considered excellent (Hallgren, 2012). The high ICC intra-rater reliabilities suggests that minimal amount of measurement error was introduced by the coding of the participants' sound files (Cicchetti, 1994).

### Order of experiment effects

There were eight different orders in which the participants completed the experiment. To determine whether the order of the experiment affected the participant's performance, a series of mixed design ANOVAs were conducted. The learning efficiency, immediate memory, and delayed memory scores were analyzed, with a 2 (listening condition: conversational vs. clear) × 2 (listen order: conversational first vs. clear first) × 2 (passage order: medipatch first vs. puffer first) × 2 (interference/filler task set order: Set A first vs. Set B first) mixed factors ANOVA, with listening condition as a within-subjects factor, and the three order variables as between-subjects factors. This was conducted for each of the dependent variables separately (see Table 5 for all *F* and *p*-values).

**Table 5:** **Order of Experimental Effects and Interactions**.

**Variables**	***F*_(1, 40)_**	***P***
**LEARN EFFICIENCY**		
Listening Condition	3.63	0.06
Listening Condition^*^Listening Order	10.68	^*^0.002
Listening Condition^*^Passage Order	0.14	0.72
Listening Condition^*^Interference Order	1.33	0.26
Listening Condition^*^Passage Order^*^Listen Order	0.87	0.36
Listening Condition^*^Passage Order^*^Interference Order	3.05	0.09
Listening Condition^*^Listen Order^*^Interference Order	0.31	0.58
Listening Condition^*^Passage Order^*^Listen Order^*^Interference Order	0.10	0.75
**IMMEDIATE MEMORY**		
Listening Condition	1.63	0.21
Listening Condition^*^Listening Order	5.91	^*^0.02
Listening Condition^*^Passage Order	2.13	0.15
Listening Condition^*^Interference Order	0.09	0.76
Listening Condition^*^Passage Order^*^Listen Order	0.63	0.43
Listening Condition^*^Passage Order^*^Interference Order	5.91	^*^0.02
Listening Condition^*^Listen Order^*^Interference Order	0.02	0.90
Listening Condition^*^Passage Order^*^Listen Order^*^Interference Order	0.00	0.95
**DELAYED MEMORY**		
Listening Condition	1.60	0.21
Listening Condition^*^Listening Order	4.04	^*^0.05
Listening Condition^*^Passage Order	0.05	0.82
Listening Condition^*^Interference Order	3.59	0.07
Listening Condition^*^Passage Order^*^Listen Order	0.40	0.53
Listening Condition^*^Passage Order^*^Interference Order	0.16	0.69
Listening Condition^*^Listen Order^*^Interference Order	0.00	1.00
Listening Condition^*^Passage Order^*^Listen Order^*^Interference Order	0.21	0.65

* *p-value bolded denotes significant*.

#### Listening condition order and listening condition interactions

**Figure 4:**
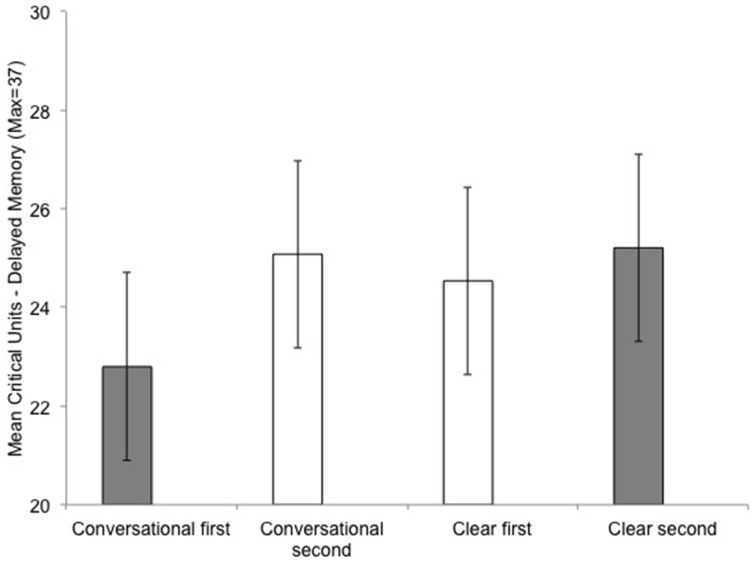
**Comparing learning effects with listening condition (conversational and clear speech) on delayed memory performance for the Quiet/Noise groups combined**. First/second indicates the order in which the participant performed that experimental listening condition. The color of the bars differentiates the between-subject listening order in which they experienced the listening condition: Gray bars represent the subgroup of participants who listened in conversation first/clear second; white bars represent the subgroup of participants who listened in clear first/conversation second. Error bars are the standard error of the mean.

There was an interaction between listening condition order (conversational-clear vs. clear-conversational) and listening condition on learning efficiency, *F*_(1, 40)_ = 10.68, *p* = 0.002, on immediate memory, *F*_(1, 40)_ = 5.91, *p* = 0.02, and on delayed memory, *F*_(1, 40)_ = 4.04, *p* = 0.05. This interaction is as follows: Performance was always better for the subgroups who experienced the listening condition as their second listening task compared to the subgroups who experienced that same listening condition as their first listening task (Figure 4).

Learning efficiency was better for second vs. first listening condition in both the conversational listening condition, *M*_first-conversational_ = 19.66, *SD* = 5.81, *M*_second-conversational_ = 21.94, *SD* = 5.40; and the clear listening condition, *M*_first-clear_ = 21.03, *SD* = 6.75, *M*_second-clear_ = 23.09, *SD* = 5.73.

Immediate memory performance was better for second vs. first listening condition in the conversational listening condition, *M*_first-conversational_ = 28.79, *SD* = 5.38, *M*_second-conversational_ = 30.42, *SD* = 4.51; and the clear listening condition, *M*_first-clear_ = 29.63, *SD* = 5.79, *M*_second-clear_ = 31.33, *SD* = 4.43.

Delayed memory was better for second vs. first listening condition in the conversational listening condition, *M*_first-conversational_ = 22.83, *SD* = 5.85, *M*_second-conversational_ = 25.08, *SD* = 6.01; and the clear listening condition, *M*_first-clear_ = 24.54, *SD* = 6.73, *M*_second-clear_ = 25.21, *SD* = 6.38.

This reflects general learning-practice effects, which were greater for the conversational (heard clear first) compared to the clear (heard conversational first) condition.

*Post-hoc* paired samples *t*-test (Bonferroni correction, alpha = 0.025) revealed that listening-order influenced the dependent variables differentially for the listening conditions. Conversational-1st order resulted in a significant difference in the two speech styles; for learning efficiency, *t*_(23)_ = 3.60, *p* = 0.002; immediate memory, *t*_(23)_ = 2.49, *p* = 0.021; and marginally significant for delayed memory, *t*_(23)_ = 1.90, *p* = 0.07. However, clear-1st order resulted in no difference in performance for listening conditions for the dependent variables, (all values for *t* < 1, *p* > 0.34). For example, when comparing the within-subject differences between the two speech styles (conversational vs. clear), there is a much smaller and non-significant differences when clear speech is heard first, where the difference between the two speech styles are significantly greater when conversational speech is heard first. Figure 4 illustrates this difference for Delayed memory performance, gray bars represent the subgroup Clear second (25.21) − Conversational 1st (22.8) = 2.41; compared to the white bars, the subgroup Clear first (25.54) − Conversational 2nd (25.08) = 0.54. This larger and significant difference between the within-subject variable (conversational vs. clear listening condition) for the Conversational-1st is evident in both learning efficiency performance, 3.43 units, compared to Clear 1st a non-significant difference of 0.91; as well for the immediate memory performance, Conversational-1st, 2.54 units, compared to Clear 1st a non-significant difference of 0.79.

As a result of these interactions between listening-order and listening condition, listening order was entered as a covariate for further hypothesis testing of learning efficiency, immediate, and delayed memory performance between the Quiet and Noise groups in the conversational and clear listening conditions.

#### Passage, interference/filler task, and listening condition interactions

There was no effect of order or interactions for passage (e.g., medipatch vs. puffer) or interference/filler task set on Learning efficiency or Delayed memory performance (see Table 5 for *F* and *p*-values). However, there was a 3-way interaction among passage (medipatch-puffer), interference/filler task (set A or B), and listening condition on immediate memory performance, *F*_(1, 40)_ = 5.91, *p* = 0.02.

The three-way interaction indicated that for the conversational speech listening conditions, those in the puffer passage with the interference task set A, immediately recalled more units, *M*_conversational/puffer-set A_ = 32.75, *SD* = 3.47, than the other 3 passage × interference task combinations, *M*_conversational/puffer-set B_ = 28.50, *SD* = 5.33, *M*_conversational/medi-set A_ = 27.67, *SD* = 5.69, *M*_conversational/medi-set B_ = 29.50, *SD* = 4.10; this was not the case in clear speech listening, the four subgroups are more similar, *M*_clear/puffer-set A_ = 31.17, *SD* = 4.11, *M*_clear/puffer-set B_ = 29.83, *SD* = 6.42, *M*_clear/medi-set A_ = 31.42, *SD* = 5.11, *M*_clear/medi-set B_ = 29.50, *SD* = 5.21.

As a result of the interactions noted above, listening condition order, passage order, and interference task order, were entered as covariates for further hypothesis testing for the differences of immediate memory between the groups (Quiet and Noise) in the conversational and clear listening conditions.

### Listening condition, competition, and interaction effects on learning and memory performance

Learning efficiency, immediate memory and delayed memory scores were analyzed with a 2 (competition: Quiet, Noise) × 2 (listening condition: conversation, clear speech) mixed design ANOVA in which listening condition was entered as the repeated measure within-subject variable and competition was a between-subject variable.

### Effects of listening condition for learning efficiency, immediate and delayed memory

There were main effects of listening condition on learning efficiency, *F*_(1, 45)_ = 13.48, *p* = 0.001, on immediate memory, *F*_(1, 43)_ = 6.35, *p* = 0.02, and on delayed memory, *F*_(1, 45)_ = 5.51, *p* = 0.02. The clear speech listening enhancements improved learning efficiency on average by 1.26 CU learned per trial and improved immediate and delayed recall on average by approximately 1 critical unit (see Table 6).

**Table 6:** **Quiet and Noise groups for Learning Efficiency, Immediate, and Delayed Memory performance in conversational and clear listening conditions**.

**Dependent variable**	**Quiet**		**Noise**		**Total**	
	***M***	***(SD)***	***M***	***(SD)***	***M***	***(SD)***
**LEARNING EFFICIENCY**						
Conversation	20.99	(6.34)	20.60	(5.03)	20.80	(5.67)
Clear	22.15	(5.92)	21.98	(6.75)	22.06	(6.28)
**IMMEDIATE MEMORY**						
Conversation	29.88	(5.15)	29.33	(4.90)	29.60	(4.90)
Clear	30.92	(5.59)	30.04	(4.80)	30.48	(5.17)
**DELAYED MEMORY**						
Conversation	24.79	(6.81)	23.13	(5.01)	23.96	(5.97)
Clear	25.00	(6.25)	24.75	(6.87)	24.88	(6.50)

*Means and Standard Deviations (CU)*.

#### Effect of the competition: Speech babble noise vs. quiet

There were no main effects of the between-subject variable (competition: noise vs. quiet) on learning efficiency, immediate memory or delayed memory (all values for *F* < 1, *p* > 0.57).

#### Interaction effects of listening condition and competition

There were no significant interactions of listening condition by competition for learning efficiency, immediate memory or delayed memory (all values for *F* < 1, *p* > 0.33). The Quiet and the Noise groups were similarly affected by the “clear” speech enhancement to the listening condition.

### Delayed memory performance and the relationship with hearing-listening and cognitive-linguistic abilities

Correlation analyses were conducted to further explore the unique contribution of the individual's hearing-listening and cognitive-linguistic abilities on delayed memory performance in the conversational and clear speech listening conditions for the two groups (Quiet and Noise) separately. The rationale to conduct this analysis for only the delayed memory performance variable was based on the following. First, all three dependent variables showed similar patterns: the clear speech technique relative to the conversational listening condition resulted in better performance for learning efficiency, immediate, and delayed memory performances (approximately one additional critical unit reported). Second, these dependent variables were significantly and highly correlated with each other (see Table 7 for correlation matrix of the dependent variables). Finally, important for the ecological validity of this study, the delayed memory variable was the metric that would support functional memory performance relevant to medical adherence.

**Table 7:** **Correlations between dependent variables for conversational (conv.) and clear listening**.

	**Delayed Conv.**	**Delayed Clear.**	**Immediate Conv.**	**Immediate Clear.**	**Learn Conv.**
Delayed Clear	0.67^**^				
Immediate Conv.	0.84^**^	0.68^**^			
Immediate Clear	0.52^**^	0.84^**^	0.49^**^		
Learn Conv.	0.84^**^	0.73^**^	0.87^**^	0.58^**^	
Learn Clear	0.62^**^	0.93^**^	0.57^**^	0.91^**^	0.65^**^

** *p < 0.01*.

The variables that reflected the hearing-listening ability as it relates to ARHL included in this analysis were LPTA4 and RPTA4, QuickSIN scores, the Hearing Handicap Inventory for Adults (HHIA), and musicianship score. The variables that reflected the cognitive-linguistic characteristics included in this analysis were as follows: auditory working memory as measured by L-span, executive function measured by verbal fluency task (FAS), lexical ability as measured by the word retrieval-picture naming task (BNT), and immediate memory as measured by the backwards digit span (Digits Back). The memory measures that were included in these correlation analyses were the delayed memory performance in the conversational and in the clear listening condition. These relationships were examined separately for the Quiet and the Noise groups.

#### Hearing-listening abilities and delayed memory performance

There were no correlations for LPTA4 and RPTA4; HHIA, QuickSIN, and Musicianship scores with delayed memory in the conversational and clear listening conditions in either the Quiet group or the Noise group when these groups are examined separately (see Tables 8, 9, 10).

**Table 8:** **Correlation analysis between delayed memory performance in the conversational (conv.) and clear listening conditions and hearing and cognitive abilities–Both groups**.

	**Delay Conv**.	**Delay Clear**	**LPTA4**	**RPTA4**	**HHIA**	**Quick SIN**	**Musician**	**L-Span**	**Digits Back**	**FAS**
Delay Clear	0.67^**^									
LPTA4	−0.17	−0.17								
RPTA4	0.06	−0.13	0.63^**^							
HHIA	−0.06	−0.07	0.56^**^	0.32^*^						
Quick SIN	−0.24	−0.27	0.23	0.24	0.18					
Musician	0.17	0.20	−0.02	0.05	−0.14	−0.45^**^				
L-Span	0.39^**^	0.28	−0.24	−0.26	−0.19	−0.26	0.28			
Digits Back	0.48^**^	0.47^**^	−0.12	−0.04	0.08	−0.48^**^	0.25	0.43^**^		
FAS	0.53^**^	0.44^**^	−0.17	−0.32^**^	−0.15	−0.27	0.20	0.52^**^	0.29	
BNT	0.56^**^	0.55^**^	−0.04	−0.05	0.04	−0.34^*^	0.18	0.30^*^	0.30	0.36^*^

* p < 0.05,

** *p < 0.01*.

**Table 9:** **Correlation analysis between delayed memory performance in the conversational (conv.) and clear listening conditions and hearing and cognitive abilities–Quiet group**.

	**Delay Conv**.	**Delay Clear**	**LPTA4**	**RPTA4**	**HHIA**	**Quick SIN**	**Musician**	**L-Span**	**Digits Back**	**FAS**
Delay Clear	0.61^**^									
LPTA4	−0.25	−0.14								
RPTA4	−0.07	−0.28	0.57^**^							
HHIA	−0.08	−0.08	0.75^**^	0.20						
Quick SIN	−0.09	−0.18	0.07	0.17	0.16					
Musician	0.05	0.22	0.13	0.27	−0.11	−0.50^*^				
L-Span	0.36	0.28	−0.23	−0.28	−0.13	−0.45^*^	0.44^*^			
Digits Back	0.44	0.20	0.19	0.27	0.04	−0.16	0.43	0.30		
FAS	0.63^**^	0.43^*^	−0.10	−0.19	−0.05	−0.31	0.09	0.43^*^	0.47^*^	
BNT	0.64^**^	0.77^**^	−0.17	−0.33	0.10	−0.29	0.21	0.39	0.54^*^	0.51^*^

* p < 0.05,

** *p < 0.01*.

**Table 10:** **Correlation analysis between delayed memory performance in the conversational (conv.) and clear listening conditions and hearing and cognitive abilities–Noise group**.

	**Delay Conv**.	**Delay Clear**	**LPTA4**	**RPTA4**	**HHIA**	**Quick SIN**	**Musician**	**L-Span**	**Digits Back**	**FAS**
Delay Clear	0.78^**^									
LPTA4	−0.08	−0.19								
RPTA4	0.24	−0.03	0.72^**^							
HHIA	−0.06	−0.06	0.33	0.49^*^						
Quick SIN	−0.34	−0.35	0.39	0.22	0.29					
Musician	0.33	0.18	−0.21	−0.08	−0.22	−0.36				
L-Span	0.44^*^	0.27	−0.25	−0.25	−0.28	−0.07	0.04			
Digits Back	0.49^*^	0.59^**^	−0.34	−0.05	0.10	−0.51^*^	0.01	0.44		
FAS	0.46^*^	0.44^*^	−0.23	−0.40	−0.27	−0.26	0.33	0.61	0.13	
BNT	0.62^**^	0.50^*^	0.03	0.08	−0.002	−0.33	0.16	0.28	0.21	0.33

* p < 0.05,

** *p < 0.01*.

However, when the entire sample was analyzed there were significant correlations with LPTA4, *r* = 0.56, *p* < 0.001; and with RPTA4, *r* = 0.32, *p* = 0.03 and self-perception of hearing handicap (HHIA); and a significant positive correlation of musicianship and listening-in-noise ability, (QuickSIN), *r* = − 0.45, *p* = 0.001. Higher musicianship scores correlated with lower QuickSIN scores or better listening-in noise abilities. This is consistent with studies that examine the relationship of degree of musicianship and perception of speech-in-noise (Parbery-Clark et al., 2009, 2012). Those with more musical training, for longer periods of time, starting at a younger age, demonstrate superior temporal processing, which supports better listening-in-noise abilities (Kraus and Chandrasekaran, 2010; Zendel and Alain, 2013). When considering the operationalized values of effect size as recommended by Cohen (1992), in which correlations >0.1 are considered small, >0.3 are considered medium, and >0.5 are considered large effect sizes. The above significant values ranged from medium to large effect sizes.

Although these hearing-listening abilities were not significantly related to delayed memory for the two listening conditions, generally the direction of the weak relationship of ARHL and memory performance was in the expected negative direction. As well, the hearing-listening measures did correlate with each other in the expected ways. For example, there were large effect sizes for the relationship between left and right acuity deficits and perception of hearing handicap (Newman et al., 1991), and a medium-large effect size of the relationship of musicianship and listening-in-noise abilities.

#### Cognitive-linguistic abilities and delayed memory performance

##### L-span: Working memory ability and delayed memory performance

There was a significant positive correlation for the L-span scores and delayed memory for the Noise group in the conversational, *r* = 0.44, *p* = 0.03, but not in the clear, *r* = 0.27, *p* = 0.20, listening condition. There were no significant correlations for the L-span scores and delayed memory performance for the Quiet group for the conversational, *r* = 0.36, *p* = 0.08, and for the clear, *r* = 0.28, *p* = 0.18 listening condition. The magnitude of the effect decreased when the listening condition was more favorable as in the clear speech without the competing noise, in which it became non-significant.

##### Backward digit spans: Short-term memory ability and delayed memory performance

In view of the fact that there were missing backward digit span scores, which most likely reflected poorer values, these results should be considered with some caution. There were significant positive correlations for the backward digit span scores and delayed memory for the Noise group in the conversational, *r* = 0.49, *p* = 0.03, and for the clear, *r* = 0.59, *p* = 0.006, listening condition. There were no significant correlations for the backward digit span scores and delayed memory performance for the Quiet group for either the conversational, *r* = 0.44, *p* = 0.06, or the clear, *r* = 0.20, *p* = 0.41, listening conditions.

When the entire sample was examined, there were significant positive correlations between backward digits spans and memory performance for both the conversational, *r* = 0.49, *p* = 0.002, and the clear, *r* = 0.47, *p* = 0.003, listening conditions. The magnitude of the effect became smaller when the listening condition was more favorable as in the clear listening or without competing noise.

##### FAS: Executive function ability and delayed memory performance

There were positive correlations of the FAS scores and delayed memory for the Noise group in the conversational, *r* = 0.46, *p* = 0.02, and for the clear, *r* = 0.44, *p* = 0.03, listening condition. There were positive correlations of the FAS scores and delayed memory for the Quiet group in the conversational, *r* = 0.63, *p* = 0.001, and the clear listening, *r* = 0.43, *p* = 0.04. The magnitude of the effect became smaller when the listening condition was more favorable in the clear speech listening condition. However, it is interesting to note that the magnitude of the relationship of executive function and delayed memory was the greatest in the Quiet group in the conversational listening condition, which is an unexpected finding that will be considered in more detail below.

##### Boston Naming Test (BNT): Lexical ability (naming/verbal fluency) and delayed memory performance

There were positive correlations for the BNT scores and delayed memory for the Noise group in the conversational, *r* = 0.62, *p* = 0.001, and the clear, *r* = 0.50, *p* = 0.01, listening condition. There were correlations for the BNT scores and delayed memory for the Quiet group in the conversational, *r* = 0.64, *p* = 0.001, and the clear, *r* = 0.77, *p* < 0.001, listening condition. The magnitude of the effect became *greater* when the listening condition was most favorable, that is in the clear speech listening condition without competing noise.

##### Summary of cognitive-linguistic abilities and delayed memory performance in the conversational and clear listening for the Quiet and Noise groups

When the entire sample was analyzed, as well as when the two groups (Quiet and Noise) were analyzed separately, there were medium to large effects of the cognitive-linguistic measures on delayed memory for the conversational and clear speech listening conditions. The magnitude of these effects generally became smaller when the listening condition was more favorable as in the Quiet group or in the clear speech enhancement (Tables 8–10).

## Discussion

The purpose of this study was to examine how auditory perception and processing of a relatively enhanced speech message (clear vs. conversational speech) affected perceptual learning efficiency, immediate, and delayed memory performance in older adults with varying levels of hearing-listening abilities. This was examined with ecologically valid methods to assess how the older adult's learning and memory performance is influenced based on real-life listening scenarios, with relevant materials and with enhancements that could be reasonably achieved.

Ultimately the research question proposed was whether ease of perceptual processing (ELU hypothesis Rönnberg et al., 2008) or effortless listening (effortfulness hypothesis, Rabbitt, 1968) mitigates the distortions from ARHL in quiet and noisy listening and promotes better learning and memory. The clear speech relative to conversational speech in this study promoted intelligibility similar to other studies that examined speech perception in younger and older adults (Ferguson, 2012). The slower rate, increased pauses, and acoustic changes (increased vowel space, F0 mean and range) enhanced the temporal-spectral aspects of the stimuli such that it was more similar to how the younger adult perceives speech compared to how the older adult typically perceives speech. Relative to younger adults with normal hearing, older adults with normal audiograms have been found to demonstrate less stable and less precise temporal processing of specific speech cues such as timing, frequency, and harmonics which interferes with speech discrimination (Anderson et al., 2012). These auditory temporal-spectral processes are necessary for discrimination of phonemes, morphemes and the regularities in the speaker's voice and speech pattern (Rosen, 1992). The stability of the acoustic information allows one to detect the regularities of the input over time. Optimal auditory perceptual ability allows one to temporally process and perceptually learn and adapt to the variability of the speaker, even within a single conversation (Mattys et al., 2012). The speech was optimized in this way to provide the older adult with the psycho-acoustic perception of speech more similar to how the younger adult experiences the stimuli (audible, slower, more distinctive).

The expectation was that clear speech would ease or decrease the effort for the experience-dependent perceptual learning of the auditory-verbal message, such that the older adult can adapt to the speaker's speech and voice pattern more efficiently, and stay attendant to the linguistic processing of the targeted message. As Salthouse (2010) states, “the most convincing evidence that the causes of a phenomenon are understood are results establishing that the phenomenon can be manipulated through interventions” (p. 157). Indeed this was the intent of the current study. Since learning and memory performance improved due to the behavioral intervention (listening enhancements) that manipulated those specific factors that were theoretically hypothesized to cause the phenomenon of poorer learning/memory performance, then these results support the hypothesis.

There are both theoretical and practical implications of these findings. Broadly defined, ARHL in older adults may indeed be contributing to age-related cognitive memory decline. Optimizing listening scenarios may significantly influence the functional performance of the older adult for IADLs.

Strengths in cognitive-linguistic abilities were positively associated with delayed memory performance with the magnitude of this effect greater in the relatively adverse listening (conversational speech). Larger effect sizes for cognitive-linguistic abilities on delayed memory performance in conversational vs. clear speech in a within-subject design suggests that indeed fewer explicit cognitive resources were required for deciphering the message in the enhanced listening.

These results are consistent with both the ELU and the effortfulness hypotheses in that making the speech audible and clearer enhanced learning and memory performance in older adults. Thus, the results of this study shed light on how sensory perception and processing declines in the older adult affect the implicit experience-dependent perceptual learning processes. This disruption to the perceptual learning processes then has cascading effects on higher-level cognitive-memory processes, delayed memory performance.

### Learning-practice effects: Order of listening condition and delayed memory performance

The significant interactions between the order of the presentation of the listening condition (conversational-clear vs. clear-conversational) and listening condition on learning efficiency, immediate and delayed memory performance in this study, are consistent with the extant literature describing a *learning-practice effect* and the related *learning curve*. A practice or learning effect is described as more positive scores (e.g., faster, more accurate, higher consistency, more efficient) with experience of task over subsequent trials of the same type of task or test. This learning-practice effect and the classic s-shaped learning curve (progress plotted on the y axis as a function of time/trials on the x axis) has been described to occur on the simplest perceptual-motor tasks as well as complex cognitive tasks (Ritter and Schooler, 2001). It is evident in educational testing, clinical neuropsychological tests, and in research with test-retest experimental designs (Hausknect et al., 2006). Learning effects may be affected by familiarity with task, decreased anxiety with repeated trials, and employment of strategies learned and transferred to the subsequent trials (Ritter et al., 2004).

The design and methods employed in this study were conducted in such a way that these learning-practice effects were anticipated (participants randomly assigned to the counterbalanced order of the variables), investigated (order effects examined); and controlled for in the analyses (entered listening-order as covariate).

### Learning-practice effect benefit on delayed memory performance

Pure listening condition effects (i.e., without learning-practice effects) can be appreciated by examining the subgroups' (*N* = 24) first listening conditions (conversation first vs. clear first). Delayed memory performance is similarly improved in clear vs. conversation in quiet (+1.5 units) and noise (+1.92 units). This supports the statistical finding of the clear speech enhancement improving delayed memory performance in quiet and noise conditions. (Figure 4).

A learning effect benefit is defined as previous experience with the task or test improving performance compared to no previous experience. It is quantified as the difference in delayed memory performance between the subgroups who had that listening condition as their second condition and the subgroups who had that same listening condition first (i.e., no prior experience with doing the experiment). For example, for delayed recall Clear 2nd − Clear 1st = +0.67; Conversational 2nd − Conversational 1st = +2.25. The reported interaction is that the learning effect benefit is differentially influenced by which listening condition was first. The benefit of experiencing the experiment first with conversational speech only increased the clear speech performance over the “pure listening condition effect” by +0.67. Where the benefit of experiencing the experiment first with clear speech increased the conversational speech performance over the “pure listening condition effect” by +2.25. In this way, conversational speech listening as the first listening condition provided less of a learning-practice effect benefit.

The learning-practice effect may be attributable to the fact that this subgroup of participants who had the second listening task as the conversational speech listening condition had the benefit of learning how to do the task first in their first listening condition (i.e., clear listening condition). They were able to *perceptually learn and adapt to* the speaker's voice and speech characteristics more easily after that first clear listening condition. Further, the finding that the magnitude of the relationship of executive function and delayed memory performance was the greatest in the Quiet group in the conversational listening condition indicates that strengths in this cognitive ability contributed to successful performance perhaps as compensation (Bäckman and Dixon, 1992; Wild et al., 2012).

These results suggest the following: (1) The “clear” speech relative to conversational speech promotes an additional perceptual learning of the speaker's voice and speech pattern, this increases the overall learning benefit even in the noise conditions, perhaps by the high perceptual load mitigating the distractor effect of the noise. (2) Conversational speech heard with ARHL decreases the learning-practice benefit, with learning-practice benefits becoming much smaller relative to the clear speech style.

### Implications

In summary, the results showed that when older adults listened to complex medical prescription instructions with “clear speech,” (presented at audible levels through insertion earphones) their learning efficiency, immediate, and delayed memory performance improved relative to their performance when they listened with a normal conversational speech rate (presented at audible levels in sound field). This better learning and memory performance for clear speech listening was maintained even in the Noise group. When the speech was manipulated so that it was sufficiently discriminable in that it could be easily segregated into meaningful units (the clear speech technique), the presence of the irrelevant distractor - speech babble noise did not differentially affect memory performance. There was a weakly associated negative relationship between ARHL and delayed memory performance in this experiment. There were medium to large positive associations between delayed memory performance and working memory, executive control and lexical abilities; however, the magnitude of these effects were larger in the conversational listening compared to the clear listening condition. This finding indicates that explicit cognitive-linguistic abilities are correlated with delayed memory performance more so in sub-optimal or adverse listening conditions. It appears that those with strengths in cognitive-linguistic abilities are able to more efficiently compensate by re-allocating resources for discrimination and comprehension of the auditory-verbal message and still have sufficient resources for the secondary task of encoding the message in memory for later recall.

Further, these results suggest that the sources of interference (speaker, listener, and environment) may interact as follows. The auditory-verbal stimuli in the conversational speech relative to clear speech listening create a demand for more cognitive-linguistic resources to achieve successful decoding of the message. As a result, the listener's limited-capacity resources are re-allocated such that fewer resources are available for learning and encoding for later recall (effortfulness hypothesis). In addition, the finding that learning-practice effects were largest when clear speech was heard first, in both quiet (+3.25) and noise (+1.25), supports the hypothesis that a high perceptual load decreases the distractor effect, where a high perceptual load spoken with *conversational* style does not (Lavie, 2005). Perhaps then when older adults listen to conversational speech rate that is further degraded by ARHL (listener source of interference), the high perceptual load does not mitigate the distractor effect (environment issues - ambient noise/reverberation/babble), which then interferes with the on-line processing of the acoustic message. Results suggest that it is this environmental issue-the distraction (even milliseconds) from the online auditory temporal-spectral processing of the message that then requires those explicit cognitive-linguistic resources to decode the message, so that fewer resources are available for encoding for later recall.

Although the data showed a main effect of listening condition (conversational and clear) on learning and memory performance, the expectation was that the competition groups (Noise vs. Quiet) would be differentially affected by the listening condition resulting in an interaction of group with listening condition. This was not found, most likely because the noise was a between group variable and there were large variances in performance within the groups. However, an interaction of listening condition order with listening condition for the subgroups of 1st vs. 2nd listening conditions was evident reflecting a perceptual learning effect or adaptation of those who listened first in the clear speech.

In addition, the expectation was that the age-related auditory acuity deficit would be more strongly correlated with learning and memory performance for the two listening conditions. The expectation was that there would be a large negative effect of hearing-listening abilities, on learning and memory performance, with the magnitude of that effect being larger in the conversational compared to the clear listening condition (as a result of the signal). Perhaps the ARHL acuity deficit was completely corrected for by presenting the stimuli at the individual's MCL. If the presentation level was set at a fixed absolute hearing level (70 dB HL) this may have then resulted in the expected negative associations of greater ARHL and poorer delayed memory performance. It also could be because the groups' PTA4 reflected normal-to-moderate hearing loss at the higher frequencies. Use of MCL presentation level for a group of older adults with more severe, precipitously-sloping high-frequency hearing loss, would not have corrected for the hearing loss as completely. Perhaps then these ARHL factors would have negatively associated with delayed memory performance.

It is probable that once the stimuli were sufficiently audible, the level of temporal-spectral degrading did not reach a threshold or tipping point in which the added distortion from ARHL interacts with the processing of the message for successful recognition and comprehension. Instead it is the cognitive-linguistic abilities that are recruited as a compensatory process for successful recognition and encoding for later recall (Bäckman and Dixon, 1992; Wild et al., 2012). The cognitive-linguistic scores significantly correlating with delayed memory performance with greater magnitudes in the conversational listening condition support this compensatory role of cognitive-linguistic abilities for adverse listening (Rudner et al., 2009).

Yet still the relative temporal-spectral manipulation of these two listening conditions might not have resulted in the conversational speech being sufficiently degraded. The temporal-spectral degrading of more typically produced conversational speech may not have been captured by this speaker's rendition. Since he was instructed to use articulation, rate and prosody for optimal clarity even for the original-conversation recording, and as a professionally trained singer and speaker, his normal conversational style is most likely comparable to citation-style speech. As Lam et al. (2012) demonstrated the instructions given to the speaker for the production of the passages affects the acoustic aspects and the intelligibility benefit (Krause and Braida, 2004, 2009; Lam et al., 2012). Citation–style speech production has been demonstrated to provide a larger intelligibility benefit than typically produced conversational speech and potentially only slightly less so from “clear speech technique” (Ferguson and Kewley-Port, 2007).

Nonetheless, enhancing the message by using a “clear speech” technique resulted in better learning and memory performance in two groups of older adults matched for age and ARHL. Additionally, the clear speech technique compared to conversational style speech reduced the negative impact that the competing noise had on learning and memory. Third, the finding that there was the largest learning effect on conversational speech as the second-listening condition after the clear speech listening condition was the first-listening condition of the experiment suggests greater perceptual learning or adaptation to the speaker's speech and voice pattern. This suggests that experience-dependent perceptual learning plays a role in facilitating or interfering with language processing and comprehension of a message and subsequent memory encoding.

### Limitations and future directions

Ecologically valid methods and stimuli are preferred for understanding complex human behaviors in the context of real life, particularly for applicability and generalizability. However, there are inherent limitations such as fewer controls of latent variables, which may confound the results. For example, relevance, familiarity, and the subjective and objective importance of instructions can influence memory performance for older adults when processing larger quantities of information (Friedman et al., 2015). The vignettes in this study were developed to be intentionally relevant, important and generally familiar (medical-patch, puffer-inhaler). However, these variables were not actively manipulated in this study. Since relevance, importance and familiarity may interact with the listening conditions, future studies should consider manipulating and/or actively controlling for these variables. It is possible that these variables influence learning and memory more so in adverse listening conditions.

Another concern was the interaction between passage order, and listening condition order on immediate memory. It is possible that one passage may have lent itself to be spoken more “clearly” than another. In the future, experiments should use a more controlled method to spectrally and temporally enhance the stimuli such as a time-expansion technique (Tun, 1998; Peelle and Wingfield, 2005). Also, to examine whether a more substantial manipulation of the temporal-spectral aspect of the stimuli interacts with ARHL, either more typically spoken conversational speech or a time-compressed technique could be employed. Additionally, using the competition as a within subject variable instead of as a between subject variable will capture the degree to which the ARHL interacts with the noise and further increases listening effort for language processing and comprehension of the message. Finally, by using a more controlled enhancement such as expanded speech in quiet this manipulation would more closely resemble the experience that the younger adult has when listening. Then younger and older participant group's learning and memory performance could be compared in the two listening conditions (time-compressed with noise and time-expanded in quiet). Those aspects that mimic ARHL should then result in poorer learning and memory performance, and those that mimic younger listening should result in better learning and memory performance for both groups. With a within-subject research design one can then examine the relationships of hearing-listening factors and cognitive-linguistic characteristics on the learning and memory performance during the two listening conditions.

### Conflict of interest statement

The authors declare that the research was conducted in the absence of any commercial or financial relationships that could be construed as a potential conflict of interest.
