# Supplementary material for: Relatively effortless listening promotes understanding and recall of medical instructions in older adults
Source: Front Psychol. 2015 Jun 9;6:778. doi: 10.3389/fpsyg.2015.00778 (PMC4460303; doi:10.3389/fpsyg.2015.00778)
Supplement: Supplementary file 1 [file DataSheet1.DOCX]

***Supplementary Material***

Relatively effortless listening promotes understanding and recall of medical instructions in older adults

**Roberta M. DiDonato_1_^1,2^*, A. M. Surprenant_2_^1^,**

^1^Cognitive Aging and Memory Lab, Memorial University of Newfoundland, Psychology, St. John’s, NL, Canada

^2^Speech Language Pathology, Medicine Department, Eastern Health, St. John’s, NL, Canada

*** Correspondence:** ^1^Cognitive Aging and Memory Lab, Memorial University of Newfoundland, Psychology, St. John’s, NL, Canada

[rmd308@mun.ca](mailto:rmd308@mun.ca)

1. **Supplementary Data**

Appendix A

**Demographic Questionnaire**

EXP# _____Participant #_________________

What is your birth date (YYYY/MM/DD)_______________________________

What is your sex? (circle one) Male Female

What is the highest level of education you have obtained?

(circle one)

Some High School High school Some College/University

College/University Graduate Some Graduate School

Graduate School/Professional degree other___________________________

What is your occupation (or, if you are retired former occupation)?________________

Please list any medications you are currently taking:

___________________________ __________________________________

__________________________________ _____________________________

Please rate your overall physical health (circle one)

Excellent Very Good Good Poor Very Poor

Do you consider yourself to be an instrumental or vocal Musician? (circle one)

YES NO

How many years have you been active in playing/singing music? _____________

How old were you when you started in music education? _____________

On average how often do you practice your music per week? ______________per week

On average how many hours do you practice per day? ___________per day.

How many languages do you speak?___________ Which languages do you speak?_______________________________________________________________________

Handedness (circle): RIGHT LEFT AMBIDEXTROUS

Appendix C

**Script of the instructions read to participants for the two listening conditions.**

The following script was printed in 16 point Cambria font, in black letters on white paper. It was read to the participant as they read along:

“Thank you for participating in this experiment.

I will explain completely at the end of the experiment why we had you listen to the type of speech samples that you heard. I will also answer any questions you have about the experiment.

For now I will explain how you will do this part of the experiment and you will have an opportunity to try a practice one.

You will hear medical prescription instructions. These instructions may sound similar to the ones you may have used in the past but these are made up.

You need to listen very carefully and try to remember each instruction, the best you can, so that you could recite back as exactly as possible in the correct order. It is not critical that you memorize the exact wording, as long as you report the gist of the instructions correctly with the critical details that are needed for these medical instructions.

For example:

If the instructions said, “place in your hand”, and you said, “put in your hand” it would be considered correct.

However, if the instructions said, “Shake the bottle and pour out two tablespoons” and you said, “Take the bottle and pour out a spoonful” it would be considered incorrect. Since “shake” is important for medication and “two tablespoons” is also important for the correct dosage needed.

Before you start the experiment we will do a practice item so that you can hear the level of loudness and the quality of the speech. The beginning part of these instructions says. What to do for aching joints. Follow these instructions carefully. Your job is to start paying very careful attention after the phrase: “Follow these instructions carefully…”. You will be asked to report back in order the instructions you heard.

Example: Training speech in noise:

Let’s try one as practice. There are just two sentences. Listen carefully to the male voice even though there are other conversations going on in the background. The male voice will say “What to do for aching joints-follow these instructions carefully…” and then he will say the two instructions that you will report back to me. Listen really carefully and report back what he says after ‘follow these instructions carefully’. “What to do for aching joints-Follow these instructions carefully…

1)

2)

The actual experiment recording will go completely through the (10) sentences without stopping. Then you will be asked to report as best you can all of what you heard and remembered immediately after each time you listen to the recording. You will have many opportunities to hear and learn these instructions. Right after each time you listen, you will report again all of what you remember in order. Some of these samples are harder to hear and understand and some are easier to hear and understand. Are you ready?”

Appendix B

**The fictional medical prescription vignettes and the practice item used in this experiment.**

Bolded items represent the critical units to recall. The bolded number at the end of the sentence represent the total # of critical units for each phrase. Sentences in italics are the carrier phrase said before each passage.

*Medipatch : This patch has a strong medication for pain and is delivered through the skin. Follow these instructions carefully.*

**Wash** your **hands _______(2)**

**Hold** the **patch** so that the **plastic backing/** **faces you ______(4)**

**Peel** off **one side** of the **plastic backing __________(3)**

**Apply** the **sticky side** **to** your **body _________(3)**

**Hold** onto the remaining piece of **plastic backing** and **pull** the patch **across** your **skin ___(5)**

To **remove** the used/old **patch** /**press** in **center** and **peel** from **edges** away from skin. __**(6)**

**Flush** the protective plastic **backing** and the used/**old patches ___(3)**

**Medicine** may **remain** **on** an old **patch** and can be **dangerous** to **children and pets ___(6)**

**Wash** your hands well **after** applying or removing a patch ___**(2)**

**Store** your **medipatches** /**out of reach** ___(**3)** ***Total units:_____/ 37***

*Puffer/inhaler: This inhaler is your rescue puffer to help you breathe easier, the capsules are used inside the puffer. Follow these instructions carefully.*

**Remove** the **blue cap** on your **rescue inhaler ___(3)**

**Hold** the inhaler at the **base** and **turn mouthpiece** in the **direction** of the **arrow ___(5)**

**Place** /**one capsule** in the **compartment** in the **base** of the inhaler **___(4)**

**Twist** the mouthpiece to the **closed position ___(2)**

**Hold** the inhaler **upright** /**squeezing** /**two** /**blue buttons** inwards to **pierce** the **capsule ___(7)**

**Breathe /out** fully, ___(2)

**Insert** the **mouthpiece into** your **mouth** and **inhale** **quickly** and **deeply ___(6)**

**Hold** your **breath** **for** a count of **ten ___(3)**

**Breathe out** /**gently** through your **mouth and nose**. **___(3)**

**Replace** the **cap ___(2) *Total units: ___/37***

*Training/ Practice: What to do for aching joints. Follow these instructions carefully.*

When you notice swelling or aching in your joints, a cold compress is helpful.

Wrap a bag of frozen peas in a dishtowel and place it on the joint.
